# Technological Innovations in Infant Formula: A Targeted Patent‐Based Analysis (2015–2025)

**DOI:** 10.1111/1750-3841.71604

**Published:** 2026-11-11

**Authors:** Giovanna Ferreira Paiva dos Santos, Maria Esther Leal da Silva Sad, Rodrigo Stephani, Ítalo Tuler Perrone, Juliana de Carvalho da Costa

**Affiliations:** ^1^ Department of Pharmaceutical Sciences Federal University of Juiz de Fora Juiz de Fora MG Brazil; ^2^ Department of Chemistry Federal University of Juiz de Fora Juiz de Fora MG Brazil

**Keywords:** bioactive compounds, infant nutrition, patent analysis, protein sources

## Abstract

The advancement of infant formulas has been driven by efforts to better replicate the nutritional and functional properties of human milk. This study aimed to analyze current innovations in infant formulas through patents published between January 2015 and December 2025, identifying key technological developments, bioactive compounds, and health claims. The analysis focused on milk‐ and whey‐based formulations intended for children aged 0 to 36 months. A search was conducted in the Espacenet database (European Patent Office, EPO), using selected international patent classification codes related to milk and whey preparations, combined with the terms “infant”, “child”, and “children”. After applying exclusion criteria, 78 patents were selected and categorized according to publication year, origin, applicants, target age group, protein source, and health claims. Results showed a predominance of Chinese patents (97%). The most frequent health claims were gut health (25%), immunity (18%), and growth (16%). Common ingredients included human milk oligosaccharides, probiotics, and fatty acids, including docosahexaenoic acid and arachidonic acid, while alternative protein sources such as goat, donkey, and yak milk were highlighted in relation to allergenicity. These findings reflect the technological trends identified within the specific patent search strategy adopted in this study. In conclusion, innovations are progressing toward mimicking breast milk effects, particularly regarding nutrition and health. However, challenges remain, including the limited bioavailability of certain compounds and the need for stronger scientific evidence supporting health claims, reinforcing the importance of further clinical studies and public policies to ensure access to safe and effective infant formulas.

## 1 Introduction

According to the World Health Organization (WHO 2022), breastfeeding is the recommended diet for babies, providing nutritional and immune support necessary for their optimal growth and health. Nevertheless, there are special conditions in which women cannot breastfeed, as when injured with an immunosuppressive disease, undergoing chemotherapy treatment, present a physical inability or due to work issues (Bick et al. 2024). In these cases, a substitute for breast milk should be sought. The compositions of infant formulas are specific to the needs of each infant, considering factors such as the age, the presence of allergies, lactose intolerance, reflux and pathologies, among others (Rodrigues et al. 2019).

Infant formulas are designed to resemble breast milk and can be prescribed in very specific medical and/or nutritional conditions (Cândido et al. 2021). According to WHO and the Food and Agriculture Organization (FAO), “Infant formula means a breast‐milk substitute specially manufactured to satisfy, by itself, the nutritional requirements of infants during the first months of life up to the introduction of appropriate complementary feeding” (WHO and FAO 2024).

With the advancement in the characterization of breast milk compounds, the market has begun to innovate and to make infant formulas more similar to human milk. Nucleotides, probiotics, prebiotics, fatty acids, and bioactive proteins are some of the compounds that are frequently added to these formulas. Each of these compounds commonly present a specific health benefit within the formulation, such as the improvement of digestion, gastrointestinal flora, immune system or vision (Almeida et al. 2021).

Patent documents have been used to investigate technological developments and innovation trends in food and nutrition‐related fields. Patent‐based analyses can provide information on technological landscapes, emerging areas of innovation, and the development of food products and formulations (Matin et al. 2024). In the field of infant nutrition, patent documents have also been used to investigate fortified infant formulas and related technological and formulation aspects (Si et al. 2026). Therefore, patent‐based analysis may provide a useful framework for characterizing technological innovations in infant formula and identifying trends in the development of formulations and their associated health claims.

Hence, the aim of this work was to investigate technological innovations identified in patent documents related to infant formula, using a targeted patent search strategy. The selected patents were categorized according to their composition, protein source, age group, and health claims. In addition, the ingredients associated with specific health claims were compared with available scientific literature.

## 2 Methodology

The patent search was made on the online database of the European Patent Office “Espacenet”, selected for its broad international coverage of patent documents and its classification‐based search functionality. The search was performed using the IPC A23C9/00 (milk preparations; milk powder or milk preparations), A23C21/04 (whey, whey preparations containing non‐milk components as source of fats and proteins) and A23C21/06 (mixtures of whey with milk products or milk components), as these classifications correspond to the milk‐ and whey‐based formulations targeted in this study. The terms “child”, “infant” and “children” were included to restrict the search to formulations intended for the target population. The search was filtered between January of 2015 to December of 2025, obtaining 186 patents.

The patents without description and claims information (12) or those that were duplicates (9) were excluded. Patents in which the title and abstract of the invention were not related to the current review (58) or only discussed product processes (8) were excluded. Subsequently, patents for children older than 36 months were also excluded (21), resulting in a total of 78 patents that were fully read and analyzed (Figure 1).

**FIGURE 1:**
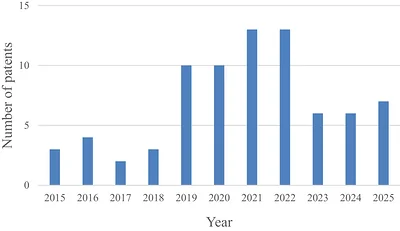
Number of patents from 2015 to 2025.

Finally, the selected patents were grouped according to their health claims: intestinal health, immunity, brain development, growth, allergy, and vision. Infant formulas that do not fit into these categories were located in the “other claims” group.

## 3 Results and Discussion

From the selected patents, relevant aspects were examined, such as publication year, countries of applicants, consumers' age, protein source, patent applicants, and health claims.

### 3.1 Publication Year

The data suggest an increasing rate of patent filings from 2019 onward (Figure 1). The highest number of filings occurred in 2021 and 2022, with 13 patents registered in each year. In 2023 and 2024, six patents were registered annually, which is lower than during the pandemic years (2019 to 2022), but higher than in the period prior to the global crisis.

The significant increase in patents, particularly between 2020 and 2022, reflects the industry's urgent response to nutritional challenges, intensified by projections of worsening conditions during the pandemic. According to the WHO and UNICEF (2021), globally, 22% of children under five years of age are stunted, meaning they fall below the expected height for their age, indicating a persistent challenge of child malnutrition. Although these data do not reflect the effects of COVID‐19, as they are based on pre‐pandemic surveys, the estimates already indicated a critical scenario that would further deteriorate with the health crisis. This context drove the demand for fortified infant formulas aimed at supporting immunity and growth.

The reduction in filings from 2023 onward may be related to the normalization of the scenario, with the resumption of public policies and nutritional programs, such as vitamin A supplementation campaigns promoted by UNICEF, which, in countries such as Bangladesh, benefited more than 193 thousand children between 2022 and 2023 (UNICEF, 2024). In addition, there has been a tightening of regulatory frameworks, with the *Codex Alimentarius* updating more stringent guidelines for substantiating nutritional claims, requiring greater technical and scientific support from companies for benefits such as “colic reduction” or “immune support” (WHO and FAO 2024). The pandemic generated innovations in record time, but the post‐crisis phase required technical consolidation and regulatory compliance in the field of infant nutrition.

By 2025, the infant formula market shows consolidation and strategic growth, driven by demand for functional nutrition. This scenario aligns with the recurrence of seven similar patents identified, indicating convergence toward optimized formulations rather than disruptive innovation. These repeated filings suggest incremental improvements, especially in products enriched with human milk oligosaccharides (HMOs), probiotics, and structured lipids, while also reflecting competitive positioning and intellectual property strategies. Thus, innovation in 2025 is increasingly characterized by refinement, regulatory alignment, and evidence‐based development.

### 3.2 Countries Applicant

According to Fortune Business Insights (2026), the global infant formula market size is projected to grow 153% in the next 5 years. Asia Pacific is a global economic hub whose infant formula market represented 63.89% in 2025 and was driven by growing female workforce participation and urbanization. China has policy reforms encouraging childbirth and rising consumer demand for advanced infant nutrition products, which may contribute to the country's strong activity in the infant formula market. There were 76 patent applications from China out of 78 evaluated. The remaining patents were from the United States (US2024008520A1) and from the World Intellectual Property Organization (WO2017030330A1). However, the targeted search strategy may have influenced the geographical distribution observed in the dataset, including the predominance of Chinese patent applications. It cannot be determined whether this pattern results from the search strategy or reflects the actual distribution of patent activity within the defined scope of the study.

### 3.3 Consumers Age

The age‐based classification of infant formulas is directly related to the specific nutritional requirements of each stage of development. From birth, the child's organism undergoes rapid physiological changes, requiring distinct compositions of proteins, lipids, carbohydrates, vitamins, and minerals. This variation justifies the regulatory classification into three main categories: infant formulas for infants (0–6 months), which serve as the exclusive source of nutrition during this stage; follow‐on formulas (6–12 months), which are complemented by the introduction of solid foods; and formulas for young children (12–36 months), indicated as a complement to the child's diet, as established by the international regulations of the *Codex Alimentarius* (WHO and FAO 2024).

In that way, infant formulas were categorized according to the consumer's age and specific needs at birth. The identified patent applications were classified according to the target consumer age specified in the patent documents. As shown in Figure 2, five categories were identified: preterm infants (7%), 0–6 months (14%), 6–12 months (4%), 12–36 months (10%), and unspecified age (65%).

**FIGURE 2:**
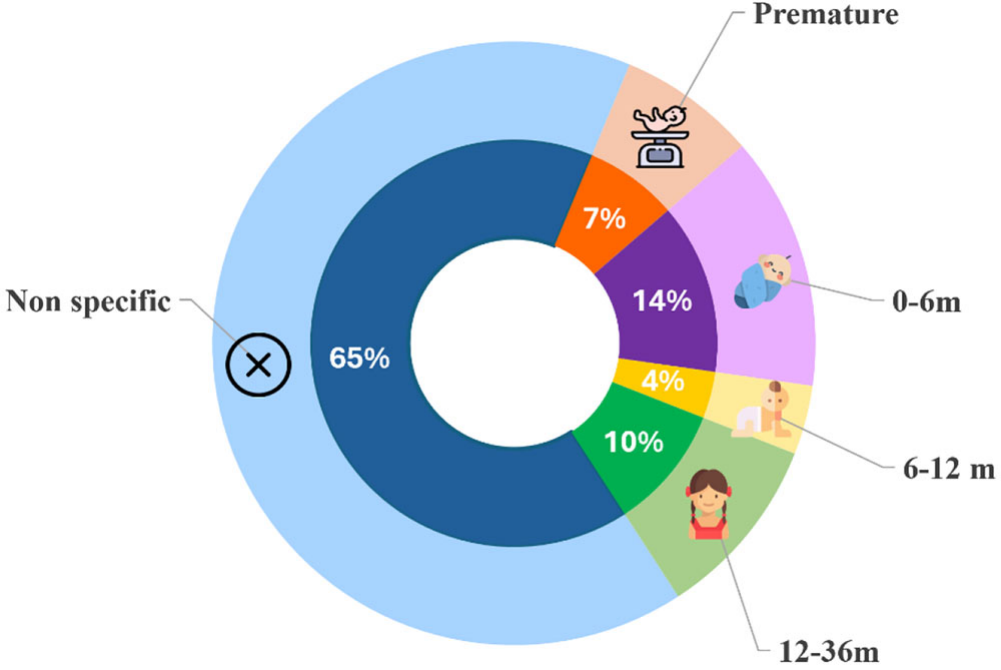
Distribution of infant formula patents according to the target age group, highlighting the predominance of non‐specific applications.

The *Codex Alimentarius* (WHO and FAO 2024) provides guidance on age differentiation: for infants (0–6 months), formulations should emphasize components such as HMOs, probiotics, and hydrolyzed proteins, with claims related to immune support and improved digestion; for age groups above 6 months, ingredients aimed at cognitive development and bone growth are highlighted, in alignment with international infant nutrition guidelines. This differentiation is essential to ensure safety and nutritional adequacy according to the stage of child development, a feature observed in the analyzed patents. Nevertheless, the emergence of multi‐stage formulas containing diverse classes of nutrients to address multiple target groups can also be observed. The distribution observed in Figure 2 indicates that the identified patents addressed different stages of early childhood, with the largest proportion not specifying a target age. Among the patents with a specified target age, formulations intended for infants aged 0–6 months represented the largest group.

### 3.4 Protein Source

Considering market trends and global demands, the choice of protein source in infant formulas is closely related to regulatory requirements, as well as to infants’ nutritional needs and parental expectations. Among the evaluated patents, cow's milk was the most frequently used protein source (*n* = 49), followed by goat's milk (*n* = 14), sheep's milk (*n* = 2), yak's milk (*n* = 1) and donkey's milk (*n* = 1). Other formulations included mixtures of milks (*n* = 6), as summarized in Figure 3.

**FIGURE 3:**
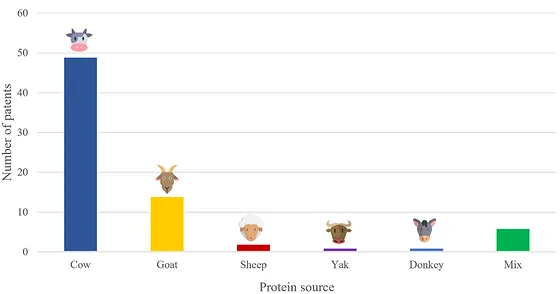
Distribution of protein sources used in Patentd infant formulas, highlighting the predominance of cow's milk and the diversity of alternative sources.

Cow's milk remains the most widely used protein source due to its broad availability and nutritional value. From a regulatory perspective, the *Codex* Standard for Infant Formula establishes requirements for the protein quality of infant formulas based on the amino acid composition of human milk. For an equivalent energy value, the formula must provide available amounts of each essential and semi‐essential amino acid at least equivalent to those in the reference protein, defined as breast milk. The standard also establishes a minimum protein content of 1.8 g/100 kcal for infant formulas based on cows’ milk protein (WHO and FAO 2024). However, cow's milk is also one of the main causes of food allergies in infants.

However, it is also one of the main causes of food allergies in infants. In this context, goat milk offers advantages: its low content of α‐s1‐casein (Prosser 2021) facilitates digestion, while the predominance of β‐casein A2 reduces allergenicity. Its unique lipid profile rich in medium‐chain triglycerides and sn‐2 palmitate mimics human lipid absorption, and its structural oligosaccharides (OSs) (especially fucosylated forms) act as prebiotics and barriers against pathogens (Van Leeuwen et al. 2020).

The milk fat globule membrane (MFGM) is a trilayer protein‐phospholipid complex that surrounds each droplet of fat secreted into milk and is present in both human and bovine milk (Hedrick et al. 2021). In addition, studies indicate that goat milk‐based formulas, that are rich in prebiotic OSs and MFGM components containing sialic acid (SA) promote a healthier gastrointestinal environment (increased *Bifidobacterium*, reduced pathogens) and strengthen immunity (modulation of pro‐inflammatory cytokines), as demonstrated in clinical trials (Gallier et al. 2020).

Other less conventional sources have also been explored, with more specific applications. Sheep milk has been studied as an alternative ingredient in infant formulas due to its distinct lipid profile. According to Casula et al. (2024), during simulated digestion, sheep milk‐based formulas showed greater release of free fatty acids and smaller lipid micelle size compared to bovine milk formulas, favoring lipase activity and suggesting enhanced digestibility. The study also identified differences in phospholipid composition, particularly sphingomyelin and phosphatidylcholine, which are known for their beneficial effects on intestinal development. Although the data indicate functional advantages, the authors emphasize that the results were obtained using an in vitro model and therefore cannot yet be recommended for infants until clinical trials are conducted (Casula et al. 2024).

Regarding donkey milk, studies demonstrate that it has a protein and lactose profile very similar to that of human milk, with a high concentration of α‐lactalbumin (also the predominant protein in human milk) and low‐molecular‐weight caseins, which form softer and more easily digestible curds. Its lipid fraction contains bioactive phospholipids, such as sphingomyelin, with potential immunomodulatory effects, as well as rare odd‐chain fatty acids with strong anti‐inflammatory activity. It also contains high levels of lysozyme and peptides similar to β‐defensins, contributing to antimicrobial protection. Studies indicate low allergenicity and good tolerance in children with cow's milk protein allergy, reinforcing its potential as a base for specialized infant formulas (Ning et al. 2023; Souroullas et al. 2018).

Thus, the diversity of protein sources in infant formulas reflects the industry's effort to provide more functional and safer alternatives, especially for populations with specific nutritional needs.

### 3.5 Patents Applicants

The proponents of infant formula patents are predominantly industrial entities. Companies account for the majority of filings, with 65 patents (83%), followed by universities with 10 patents (13%). Collaborative efforts between companies and universities represent a smaller proportion, with 2 patents (3%), while only one patent was filed by an individual, as shown in Figure 4.

**FIGURE 4:**
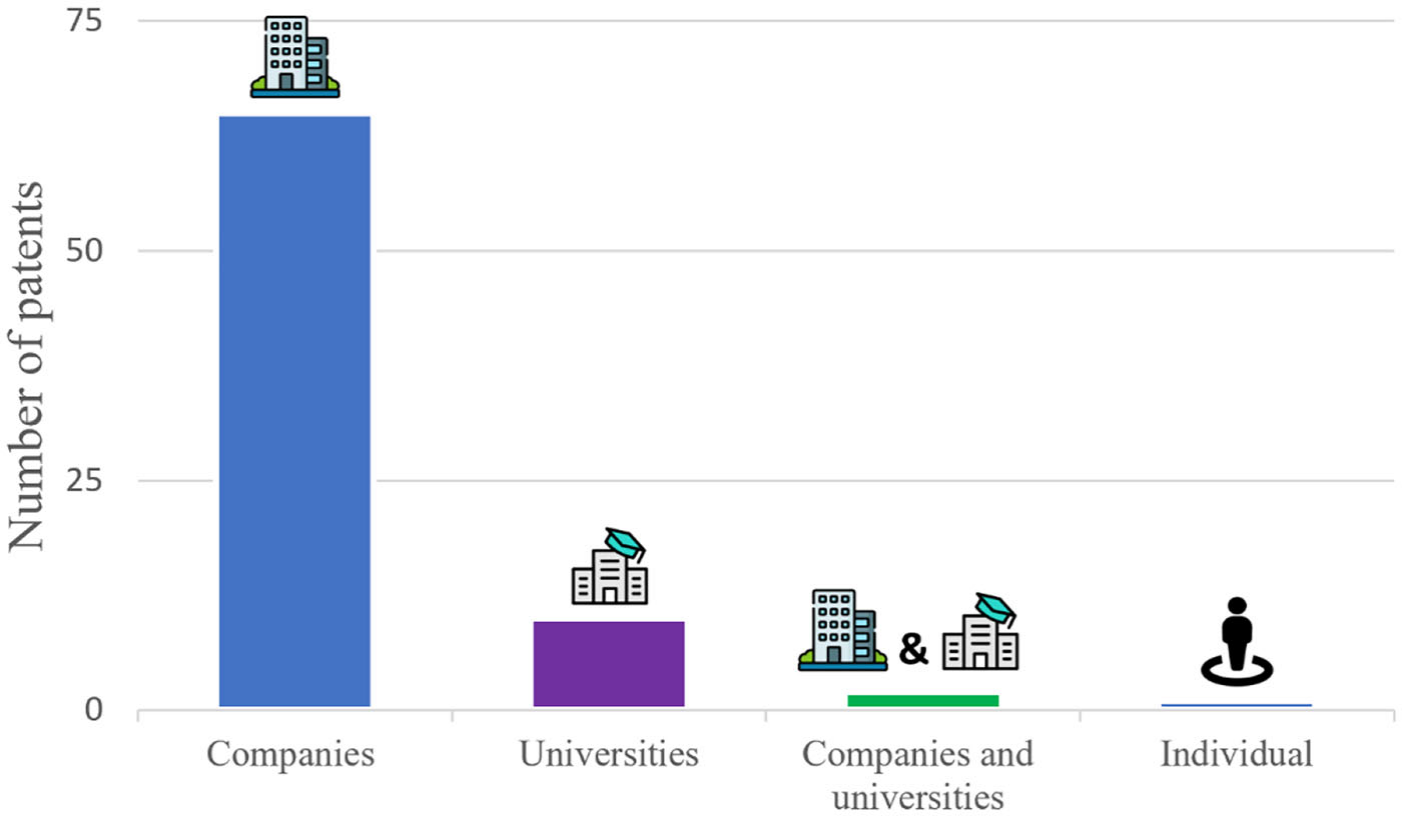
Distribution of patent applicants in infant formula technologies, emphasizing the leading role of companies and the contribution of academic institutions and collaborative efforts.

Among the industrial entities, prominent contributors include INNER MONGOLIA YILI INDUSTRIAL GROUP, with 11 patents; SHIJIAZHUANG JUNLEBAO DAIRY CO., LTD., with 8 patents; BEIAN YEEPER NUKA DAIRY INDUSTRY CO., LTD., with 4 patents; and AUSNUTRIA DAIRY (CHINA) CO., LTD. and BRIGHT DAIRY & FOOD CO., LTD., each with 3 patents. Although it was not possible to verify whether each patent resulted in a specific commercial product due to language limitations, these findings suggest that such companies offer a diverse portfolio of infant formulas to meet different market demands, based on the search results in this study.

### 3.6 Claims

Patents were classified according to their health claims, as summarized in Figure 5. Some patents presented more than one claimed benefit; therefore, the total number of identified claims exceeds the total number of patents evaluated. According to Table 1, infant formula patents were categorized based on their composition, protein form and source, target age group, and claimed benefits.

**FIGURE 5:**
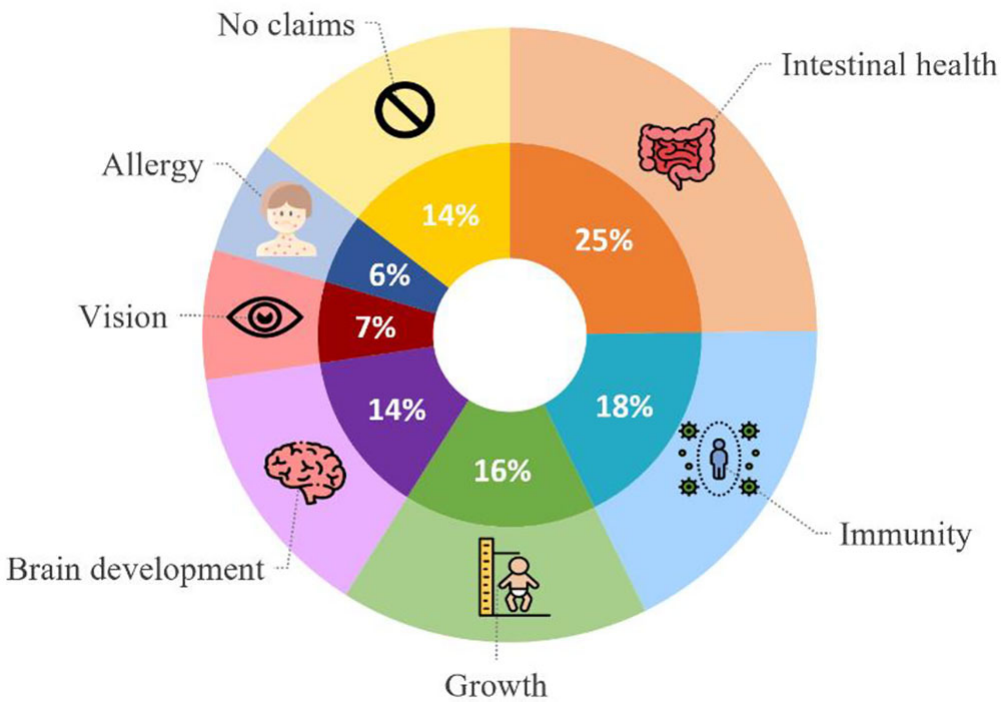
Distribution of health claims in Patentd infant formulas.

**TABLE 1:** Infant formula patent´s compounds, proteins form and source, target age group, and claimed benefits.

#	Patent number	Compounds	Protein form	Protein source	Age	Claims	Ref.
1	CN115500398A	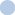 Fructooligosaccharide 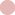 β‐casein	Raw milk Demineralized whey powder	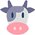	NS	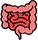	(Jiang et al. 2022)
2	CN104489132A	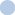 Lactulose	Milk powder and/or milk Desalted whey powder	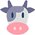	NS	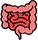	(Jia et al. 2015)
3	CN106578134A	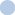 OSs 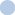 Salicylic acid 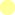 OPO 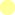 DHA 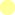 ARA 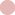 LA	Desalted whey powder MFGM Proteins	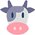	NS	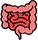	(Liu et al. 2017)
4	CN113973926A	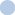 Sugar 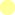 Fat 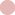 Hydrolyzed protein	Yak milk Fresh milk Demineralized whey protein powder Whey powder Whey protein concentrate	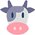 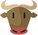	NS	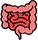	(Ma et al. 2022)
5	CN110754538A	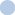 Fructooligosaccharide 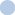 Galactooligosaccharides 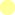 DHA 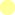 ARA	Skim milk powder Whole milk powder Whey powder Whey protein powder	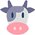	NS	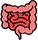	(Wang 2020)
6	CN120323525A	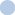 Lactose 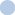 Galactooligosaccharides 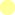 Vegetable blending oil 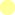 DHA 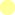 ARA 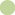 Bifidobacteria	Raw milk Whole milk powder Skim milk powder Whey protein powder Desalted whey powder	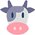 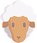	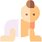	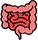	(Gao et al. 2025)
7	CN119453324A	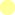 DHA 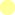 Eicosatetraenoic acid 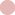 1,3‐dioleoyl‐2‐ palmitoyltriglyceride 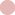 Nucleotides 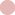 LA 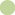 Probiotics	Raw milk Whole milk powder Skimmed milk powder Whey protein powder Desalted whey powder	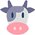	NS	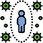	(Liu et al. 2025)
8	CN119234885A	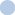 Lactose 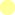 Vegetable blended oil 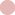 Casein	Whey protein	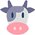	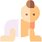	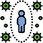	(Zhou et al. 2025)
9	CN113796424A	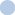 Oligomer isomaltose 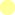 ARA 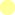 DHA 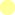 N‐acetylneuraminic acid 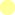 Phosphatidylserine	Sheep milk powder demineralized whey powder whole sheep milk powder	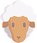	NS	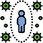	(Meng et al. 2021)
10	CN110074209A	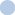 HMOs 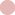 LA	Desalted whey powder skim milk powder concentrated whey protein	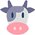	NS	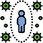	(Li et al. 2019)
11	CN119453323A	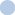 Lactose 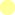 Vegetable blending oil 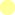 Phospholipids 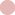 Casein 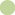 Prebiotics	Raw milk Desalted whey powder Skim milk powder Whey protein powder	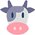	NS	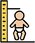	(Li et al. 2025)
12	CN111616232A	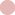 Casein phosphopeptide 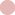 Hydrolyzed egg yolk 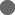 Calcium, vitamin K2	Milk or goat milk Skimmed milk powder Desalted whey powder	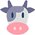 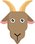	NS	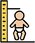	(Yuan et al. 2020)
13	CN113647469A	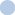 Polyisomaltose 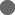 Vitamins and minerals complex	Whole milk powder demineralized whey powder skimmed milk powder	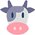	NS	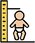	(Tang 2021)
14	CN109527116A	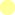 Triglyceride powder 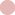 Hydrolyzed whey protein	Hydrolyzed whey protein powder	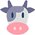	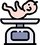	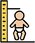	(Ma et al. 2019)
15	CN117481216A	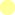 Gamma‐aminobutyric acid	Whole milk powder Skimmed milk powder	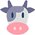	NS	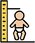	(Wang et al. 2024)
16	CN115024364A	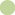 *Bifidobacterium animalis* BB‐12 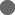 Vitamins and minerals complex	Raw goat milk Goat demineralized whey powder	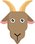	NS	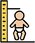	(Zhao et al. 2022)
17	CN113575695A	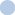 Fructooligosaccharide 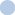 Galactooligosaccharides 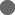 Vitamins and minerals complex	Whole milk powder Desalted whey powder	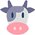	NS	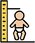	(Li and Jiang 2021)
18	CN117397816A CN117397816B	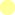 OPO 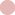 Hydrolyzed protein 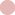 Nucleotides	Hydrolyzed milk protein Hydrolyzed whey protein.	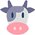	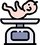	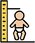	(Li et al. 2024)
19	CN119632105A	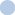 Lactose 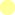 Phospholipids 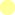 Vegetable blending oil 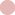 Casein 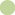 Prebiotics	Raw milk Skim milk powder Whey protein powder Desalted whey powder	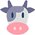	NS	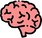	(Li et al. 2025)
20	CN111011541A	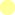 N‐acetylneuraminic acid 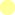 Phosphatidylserine 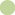 *Bifidobacterium animalis* BB12	Goat milk Goat whey powder Whey protein concentrate	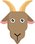	NS	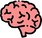	(Pan 2020)
21	CN117652577A	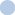 SA 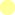 Sphingomyelin 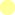 Phosphatidylcholine	Desalted whey powder Whey protein concentrate powder	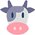	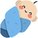 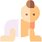 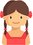	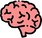	(Gao et al. 2024)
22	CN112655774A	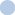 3'‐sialyllactose 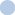 Salicylic acid 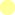 1,3‐dioleic acid‐2‐palmitic acid triglyceride 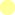 Gangliosides 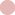 Nucleotides	Whole fat cow milk Cow demineralized whey powder	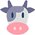	NS	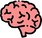	(Feng et al. 2021)
23	CN114668049A	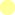 Ganglioside easy to digest and absorb	Whole fat sheep milk Sheep demineralized whey powder	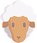	NS	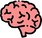	(Duan and Huang 2022)
24	CN111616234A	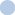 Fructooligosaccharide 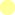 DHA 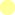 ARA powder	Skimmed milk powder Desalted whey powder	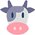	NS	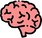	(Li 2020b)
25	US2024008520A1	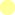 Sphingomyelin	Whey protein concentrate from milk	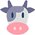	NS	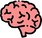	(Alvey et al. 2024)
26	CN121040530A	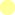 Anhydrous butter 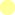 Mixed vegetable fat 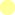 DHA 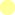 ARA	Raw milk Whey powder	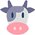	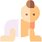 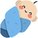 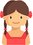	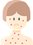	(Ma et al. 2025)
27	CN107047801A CN107047801B	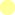 Eicosatetraenoic acid 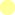 DHA	Demineralized whey powder Dried skim milk whole milk powder Concentrated whey protein powder Hydrolyzed whey protein powder	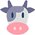	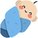 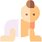 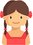	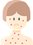	(S. Liu and Shen 2017)
28	CN114009510A	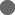 Vitamins and minerals complex	Raw milk desalted goat Whey powder hydrolyzed goat Whey protein powder	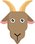	NS	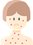	(Ma et al. 2022)
29	CN112167360A	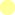 OPO 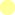 ARA powder 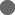 Vitamins and minerals complex	Raw goat milk High‐oil desalted goat whey powder	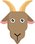	NS	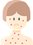	(Wei et al. 2021)
30	CN110999984A	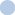 Galactooligosaccharides 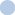 Polyfructose 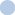 Fructooligosaccharide 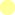 ARA powder 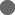 Vitamins and minerals complex	Milk powder Concentrated whey protein powder	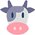	NS	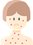	(Wang et al. 2020)
31	CN108967557A	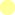 Mono‐diglyceride fatty acid ester 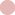 Casein 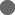 Vitamins and minerals complex (Potassium citrate)	Hydrolyzed desalted whey powder Hydrolyzed skimmed milk powder	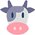	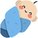	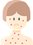	(Jiang et al. 2018)
32	CN120203136A	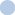 Lactose 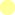 Galactooligosaccharides 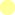 Fructooligosaccharide 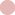 Vegetable blending oil 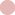 Phospholipids 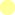 DHA 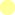 ARA LA Nucleotides	Raw milk Whole milk powder Skim milk powder Whey protein powder Desalted whey powder	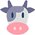 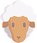	NS	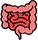 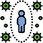	(Liu et al. 2025)
33	CN109845827A	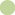 *Bifidobacterium animalis* BB‐12	Desalted whey powder Whole milk powder Concentrated whey protein powder	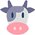	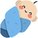	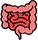 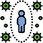	(Zeng 2019a)
34	CN106212723A	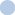 Fructooligosaccharide 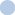 Galactooligosaccharides 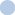 Polyfructose 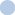 Polydextrose	Whey protein powder Hydrolyzed milk protein	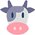	NS	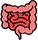 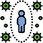	(Ouyang et al. 2016)
35	CN112075504A	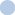 Fructooligosaccharide 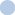 Galactooligosaccharides 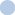 Ganglioside 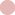 Osteopontin 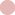 LA 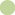 *Lactobacillus paracasei* N1115	Partially enzymatically hydrolyzed desalted whey powder	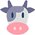	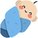	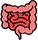 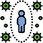	(Feng et al. 2020)
36	CN112790250A	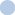 2'‐fucosyllactose Lactose‐N‐tetraose 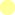 Triglyceride 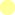 OPO 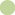 *Bifidobacterium animalis* subsp. lactis i797	Cow milk/goat milk Desalted whey powder Concentrated whey protein powder	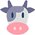 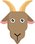	NS	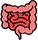 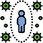	(Wei et al. 2021)
37	CN115067396A	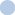 Fructooligosaccharide 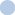 Galactooligosaccharides 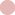 LA	Raw milk Demineralized whey powder Whey protein powder Alpha‐whey protein MFGM protein	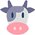	NS	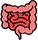 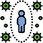	(Chen et al. 2022)
38	CN114176137A	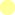 ARA 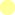 DHA 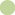 *Bifidobacterium lactis* (Bi‐07/HN019)	Whey protein concentrate powder	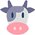	NS	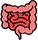 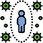	(Zhang 2022)
39	CN107927198A	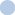 Polyfructose 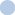 Fructooligosaccharides 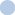 Galactooligosaccharides 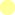 Chain triglyceride 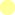 DHA 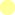 ARA	Whey protein powder	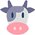	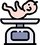 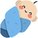	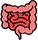 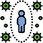	(Chen et al. 2018)
40	CN107347998A	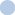 HMOs 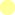 OPO 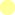 Soybean phospholipid 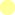 ARA 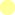 DHA	Desalinated whey powder Nonfat dry milk powder Whey protein concentrate Bovine whey polypeptides or goat whey polypeptides	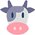 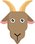	NS	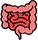 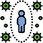	(Zhang et al. 2017)
41	CN110122589A	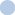 Fructooligosaccharides 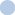 Galactooligosaccharides 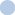 Polydextrose 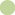 Probiotic microcapsule	Concentrated whey protein LA Hydrolyzed whey protein powder	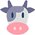	NS	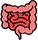 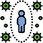	(Tang and Xia 2019)
42	CN114223728A CN114223728B	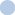 HMOs 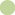 *Bifidobacterium longum* subsp. *infantis*	Milk powder Whey protein	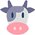	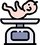	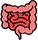 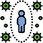	(An et al. 2022)
43	CN115736026A	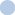 Fructooligosaccharides 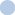 Galactooligosaccharides 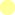 Medium chain triglycerides 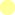 Milk fat 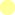 OPO 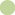 *Bifidobacterium longum* subsp. *infantis* YLGB‐1496	Raw milk Whey protein powder Desalted whey powder alfa‐lactalbumin powder beta‐casein powder Raw milk Whole milk powder Skimmed milk powder	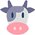	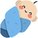 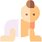 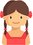	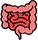 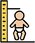	(Zhao et al. 2023)
44	CN105746728A	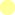 OPO 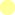 DHA 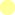 Eicosatetraenoic acid 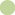 *Bifidobacterium animalis* (BB‐12) 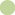 *Bifidobacterium lactis* (Bi‐07)	Skim milk powder Desalted whey powder Concentrated whey protein powder	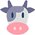	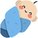	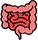 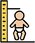	(Tu et al. 2016)
45	CN113180117A	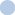 Oligomers 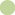 Probiotics	Whole milk powder Skimmed milk powder Demineralized whey powder	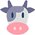	NS	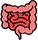 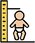	(Gao et al. 2021)
46	CN104351356A	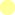 OPO 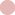 LA	Hydrolyzed whey protein Hydrolyzed casein Concentrated whey protein	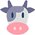	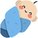	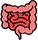 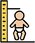	(Mo et al. 2015)
47	CN112535218A	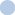 HMOs 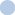 Fruit powder 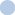 Salicylic acid 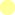 OPO 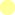 ARA 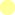 DHA algae oil 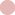 LA 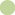 Probiotic	Cow milk/goat milk Desalted whey powder Whey protein powder	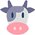 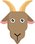	NS	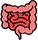 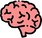	(Mo et al. 2015)
48	WO2017030330A1	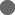 Vitamins and minerals complex	Milk powder	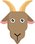	NS	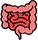 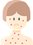	(Kim et al. 2017)
49	CN112889930A	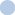 Fructooligosaccharide	Whole fat goat milk Whey powder Concentrated whey protein powder	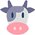 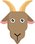	NS	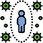 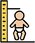	(Xiao et al. 2021a)
50		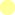 ARA 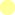 DHA 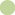 *Bifidobacterium* BB‐12 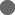 Zinc, iron, iodine, copper, manganese, selenium, and calcium	Skimmed milk powder desalted whey powder	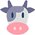	NS	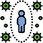 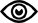	(P. Li 2020)
51	CN117481200A	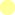 Medium chain triglyceride 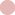 Choline	Hydrolyzed milk protein Hydrolyzed whey protein.	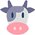	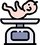	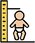 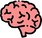	(Li et al. 2024)
52	CN109845826A CN109845826B	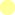 Walnut fat powder 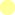 OPO 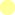 DHA 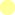 ARA	Milk powder Demineralized whey powder	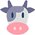	NS	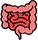 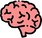 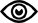	(Zeng 2019b)
53	CN116268113A	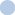 HMOs 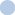 Salicylic acid 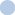 2'‐fucosyllactose 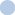 3‐fucosyllactose	Raw milk Desalted whey powder Enzyme hydrolyzed whey powder Αlpha‐whey powder	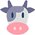	NS	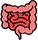 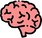 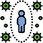	(Li et al. 2023)
54	CN117044790A	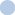 Fructooligosaccharide 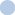 Polydextrose 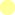 DHA 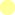 ARA 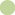 *Bifidobacterium animalis* BB‐12	Goat milk powder Desalted goat whey powder	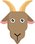	NS	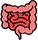 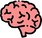 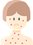	(Li and Li 2023)
55	CN112889930A	NA	Whole fat goat milk Whey powder and protein powder	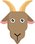	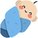 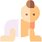 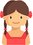	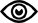 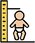 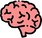	(Xiao et al. 2021b)
56	CN115644259A	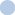 Fructooligosaccharides 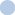 Galactooligosaccharides 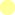 DHA 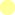 ARA	Raw goat milk Bovine desalted whey powder	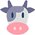 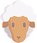	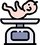 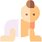	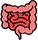 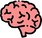 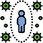 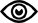	(Li et al. 2023)
57	CN114431304A	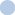 Fructooligosaccharides 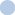 Galactooligosaccharides	Raw donkey milk Milk fat globule protein	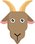	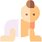	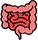 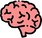 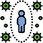 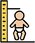	(Li et al. 2023)
58	CN110037125A	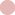 Peptide product obtained by adding fresh yak milk and a fermentation liquid of *Bacillus subtilis*	Yak milk Fresh milk Demineralized whey protein powder Whey powder Whey protein concentrate	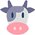 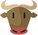	NS	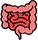 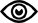 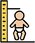 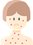	(Li et al. 2019)
59	CN113841743A	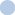 Fructooligosaccharides 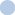 Galactooligosaccharides	Full‐cream goat milk powder Desalted goat whey powder	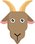	NS	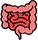 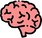 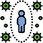 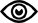 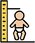	(Liu et al. 2021)
60	CN109221443A	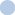 Fructooligosaccharides 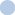 Galactooligosaccharides 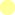 OPO, Palmitate, Inositol 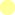 DHA 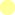 ARA 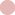 Taurine	Whole goat milk powder Desalted whey powder Whey protein concentrate powder	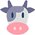 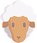	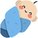	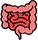 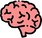 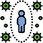 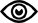 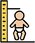	(Ma et al. 2019)
61	CN105831284A	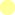 DHA 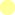 ARA 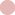 LA	Concentrated hydrolyzed whey protein powder	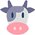	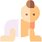	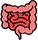 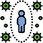 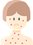 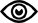 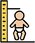	(Liu et al. 2016)

Compounds		Protein source		Age		Claims	
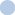	Carbohydrates	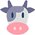	Cow	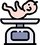	Premature	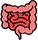	Intestinal
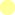	Lipids	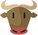	Yak	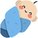	0–6 months	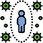	Immunity
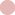	Proteins	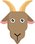	Goat	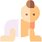	6–12 months	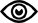	Vision
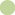	Probiotics	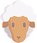	Sheep	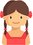	12–36 months	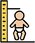	Growth
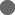	Vitamins and minerals					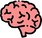	Brain
						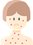	Allergy

*Note*: Legend: NS—not specified. The first letters of each patent number indicate the country or organization of origin (e.g., CN: China; US: United States; WO: World Intellectual Property Organization).

Patents related to intestinal health represent the largest category (25%), encompassing formulations designed to improve nutrient absorption, support the intestinal microbiota, enhance intestinal transit, and soften stools. The immunity category accounts for 18% of the claims and includes patents aimed at promoting the maturation of the infant immune system and enhancing immune function.

The growth category (16%) comprises patents associated with improved calcium absorption and overall infant development. The categories brain development, vision, and allergy correspond to 14%, 7%, and 6% of the claims, respectively. Patents classified under brain development focus on enhancing cognitive functions and supporting neurodevelopment, while the vision category includes formulations that promote visual development. The allergy category encompasses patents developed for infants with milk protein allergies.

Finally, the “other claims” category (14%) includes patents without a specific functional claim, which are detailed in Table 2.

**TABLE 2:** Patents with no claims.

#	Patent number	Compounds	Protein	Protein source	Age	Ref.
1	CN114052085A CN114052085B	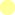 OPO	Demineralized whey powder Whole milk powder	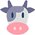	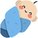	(Liu et al. 2022)
2	CN114258961A	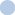 MOs 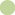 *Bifidobacterium animalis*	Whey protein	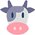	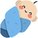 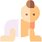 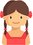	(An et al. 2022)
3	CN116138322A	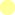 Fat globule membrane 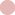 Casein	Demineralized whey powder Whole milk powder	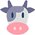 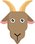	NS	(Qu et al. 2023)
4	CN105010573A	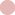 Condensed whey albumin 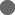 Vitamins and minerals	Whole milk powder or milk Desalinated whey powder	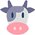	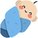 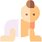 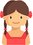	(Wang 2015)
5	CN108651634A	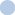 Galactooligosaccharides 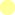 DHA oil 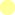 ARA 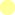 Linoleic acid alpha‐linolenic 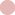 Casein hydrolysate	Desalted whey powder Skimmed milk Whey protein concentrate	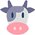	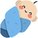	(Qi 2018)
6	CN112535217A	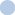 Milk oligosaccharide 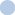 Phospholipid 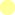 OPO 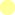 Acid triglyceride powder	Parts of desalted whey powder Concentrated whey protein powder	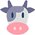 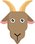	NS	(Xun et al. 2021)
7	CN110100905A	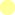 OPO	Desalted whey Powder concentrated Whey protein powder	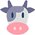	NS	(Li et al. 2019)
8	CN110742130A	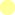 OPO 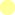 Lipidosome	Goat milk powder	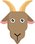	NS	(Li et al. 2020)
9	CN105994680A	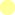 OPO 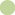 Probiotics 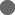 Vitamins and minerals	Whole fat goat milk powder Desalted goat whey dry product	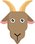	NS	(Mou et al. 2016)
10	CN115005284A CN115005284B	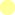 OPO	Goat milk powder	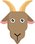	NS	(Chi et al. 2022)
11	CN111357829A	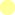 Part of citric acid 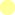 L‐Sodium ascorbate	Full‐cream raw milk Desalted whey powders	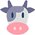	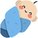 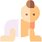 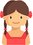	(Jean 2020)
12	CN112998080A	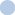 Egg yolk phospholipid 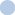 Soybean phospholipid 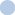 Milk phospholipid	Raw milk or raw goat milk Demineralized whey powder Hydrolyzed whey protein powder	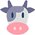 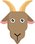	NS	(Yuan et al. 2021)
13	CN110074188A	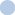 HMOs	Whole milk powder Desalted whey powder	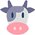	NS	(Wang et al. 2019)
14	CN112602783A	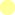 Egg yolk lecithin 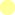 Vegetable oil	Desalted whey powder Whey protein	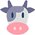	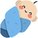	(Qi et al. 2021)
15	CN110477129A	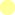 MFGM	Desalted whey powder Whey protein	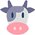	NS	(Li 2019)
16	CN114208895A	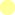 Edible plant blend oil 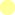 Breast milk substitute fat	Cow milk Demineralized whey powder Concentrated whey protein powder	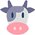	NS	(Jie et al. 2022a)
17	CN114208896A	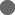 Buffer salt (sodium citrate)	Raw milk Demineralized whey powder	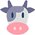	NS	(Jie et al. 2022b)

Compounds		Protein source		Age		Claims	
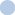	Carbohydrates	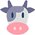	Cow	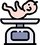	Premature	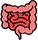	Intestinal
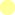	Lipids	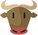	Yak	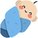	0–6 months	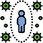	Immunity
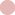	Proteins	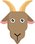	Goat	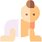	6–12 months	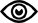	Vision
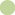	Probiotics	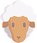	Sheep	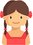	12–36 months	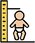	Growth
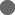	Vitamins and minerals					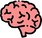	Brain
						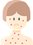	Allergy

*Note*: Legend: NS—not specified. The first letters of each patent number indicate the country or organization of origin (e.g., CN: China; US: United States; WO: World Intellectual Property Organization).

### 3.7 Intestinal Health

The composition of breast milk plays a crucial role in the early development of infant intestinal health, influencing nutrient absorption, immune system maturation, and overall physiological well‐being and growth. Breast milk provides a complex array of bioactive components such as prebiotics (primarily OSs), probiotics (including *Bifidobacterium* and *Lactobacillus* spp., *Streptococcus*, *Staphylococcus*, *Ralstonia*, *Bacteroides*, *Enterobacter*, and *Enterococcus*), and immunoglobulins, essential for establishing a diverse and stable intestinal microbiota (Carr et al. 2021).

Additionally, breast milk shapes the intestinal microbiota of newborns, a process influenced by factors such as gestational age, mode of delivery, maternal weight, stress, and diet, which significantly impact the relative proportion and establishment of bacteria, digestive maturation, and immune response (Ferreres‐Serafini et al. 2024). Charton et al. (2022) indicates that the intestinal microbiota plays a crucial role in overall well‐being, influencing not only gastrointestinal disorders but also complex interactions with other body systems, such as the gut‐brain axis, and studies related to the gut microbiota influence reducing the risk of developing some neuropsychiatric or neurodevelopmental diseases. Thus, it can be firmly concluded from scientific literature that cultivating a robust microbiota in infants primarily through feeding profoundly influences overall bodily health.

Infant formulas aim to replicate the nutritional and immunological benefits of breast milk, and ongoing innovations in infant formula formulations continually strive to improve nutritional composition, mimic immunological benefits, and support brain development, recognizing the importance of intestinal health beyond the gastrointestinal tract (Ferreres‐Serafini et al. 2024).

The significant presence of bifidobacteria in the microbiome of children up to 16 months suggests a microbiota similar to that found in infants, as adults have low concentrations of these bacteria. Based on these observations, infant formulas designed for children up to 16 months should not only include bifidobacteria but also incorporate substances in their formulations that promote their growth and establishment in the gut microbiota, such as prebiotic oligosaccharides (Samarra et al. 2025). Therefore, formulating infant formulas with specific nutrients and prebiotics that stimulate the growth of bifidobacteria may be essential to mimic the microbiota composition found in healthy infants during this critical developmental period and potentially mitigate diseases associated with microbial imbalance later in life (Adamczak et al. 2024).

Food intolerance is also a critical issue in infant nutrition, as it can cause various gastrointestinal symptoms such as colic and diarrhea, disrupting the microbiota and compromising healthy development (Khan et al. 2026). Recent studies have suggested that plant‐based beverages or alternative milks in formulas can serve as viable alternatives for babies who cannot consume breast milk or cow's milk due to intolerances (Odusanya et al. 2022). These options are already utilized by adults with this condition, offering a promising alternative to ensure a balanced and adequate diet from the early months of life. Recent studies have explored diverse alternatives in infant formula, targeting individuals with food intolerances. Ramirez‐Farias et al. (2024) emphasize that rice‐based formulas offer a nutritious option for children with food intolerances. Darma et al. (2024) suggest that soy‐based formulas are promising as substitutes for cow's milk. Additionally, Bahbah et al. (2022) highlight the widespread use of goat milk in infant formulas across the Middle East and North Africa, where 65% of healthcare professionals involved in infant feeding, such as pediatricians, nutritionists, and pharmacists, were aware of nutritionally adapted goat milk formula as an alternative to cow dairy products, and 37% would recommend its routine use in infants under 24 months. Furthermore, Shrestha et al. (2021) investigated the gastrointestinal tolerance of sheep milk in women, underscoring the need for further research to evaluate its suitability for infant nutrition.

Despite advances in incorporating components like microorganisms, OSs, and essential fatty acids to promote intestinal microbiota development, the complexity of this interaction remains a significant challenge in fully replicating the benefits of breast milk in infant formulas. Due to this demand, innovations in formulas aiming to positively impact intestinal health are ongoing, with new patents filed annually (Kariyawasam et al. 2025).

It can be observed that 25% of patents include a claim related to intestinal health, thus being the greatest appeal of infant formulas, highlighting the relevance of gastrointestinal function for infant health, as well as the broader implications of intestinal health for immunity and physical and cognitive development. Moreover, among patents exclusively focusing on this claim, the presence of carbohydrates acting as a prebiotic, particularly oligosaccharides known for their easy digestibility, is notable. These findings emphasize the importance of a balanced microbiota for infant health. Additionally, patents often feature 1,3‐dioleoyl‐2‐palmitoyltriglyceride (OPO), docosahexaenoic acid (DHA), and arachidonic acid (ARA), compounds naturally present in breast milk that contribute to infant nutrition, growth, cognitive development, vision, and establishment of the infant's microbiota (Borewicz et al. 2024; Ni et al. 2024).

When claims related to intestinal health are associated with immune benefits, probiotic microorganisms such as *Bifidobacterium animalis*, *Lactobacillus paracasei*, and *Bifidobacterium lactis* are frequently incorporated, as they contribute to the modulation of a healthy gut microbiota, which is directly linked to immune function, as previously discussed. HMOs are frequently employed to enhance formula tolerance, providing better intestinal comfort and mitigating allergic reactions. Claims related to growth and brain development introduce compounds like SA and lactoferrin (LA), with LA being a glycoprotein rich in SA, both acting in cognitive, intestinal, and immune functions in infants (Wang 2016; Wang et al. 2024).

#### 3.7.1 Immunity

The immune system of infants begins to develop gradually through exposure to pathogens. Meanwhile, they rely on passive immunization provided by antibodies present in breast milk, a particularly urgent demand because their defense mechanisms are still naïve, making them highly susceptible to a variety of diseases (Carr et al. 2021). Vassilopoulou et al. (2024) report that exclusive breastfeeding significantly reduces hospitalization and mortality in infants with acute respiratory infections, as breast milk is crucial for infant immunity, providing immunoglobulins, leukocytes, antimicrobial peptides, and other immunomodulatory factors.

The presence of these immune factors from breast milk not only directly combats invading microorganisms but also strengthens the three gastrointestinal barriers: mucus production, which prevents invaders from adhering to the intestinal wall; secretion of antimicrobial peptides; and production of IgA (Al‐Beltagi 2025). Breastfeeding significantly reduces, for example, the risk of diarrhea and sepsis in infants, especially in developing countries, due to the presence of IgA specific to enterotoxic species of bacteria such as *Escherichia coli* and *Campylobacter*, significant pathogens in this context (Abbas et al. 2022). It is also important to note that the presence of prebiotics and probiotics strengthens the developing microbiota in the gastrointestinal tract of these children, competing with any microorganism seeking to colonize via this route (Carr et al. 2021). These three gastrointestinal barriers are dependent on the integrity of the epithelial barrier, and some studies, such as Wieser et al. (2024), explore the relationship between compounds that protect the epithelial barrier and the immune system. Therefore, intestinal health plays a crucial role in infant immunity, as discussed in the topic of intestinal health.

Infant formulas aiming to replicate as much as possible the benefits of breast milk on immunity often add components that strengthen the gastrointestinal epithelial barrier, such as LA, prebiotics, and probiotics (Wang et al. 2024). LA, for instance, is a multifunctional protein that primarily acts on immunity development and early establishment of the intestinal microbiota, while probiotics like *Bifidobacterium* and *Lactobacillus* promote a healthy intestinal microbiome (Ferreres‐Serafini et al. 2024). Any other added substance influencing the balance of microbiota, such as OSs, will contribute to infant immunity development.

The immunity claim comprises 18% of the total, making it the second most utilized claim in patent applications for infant formulas. Despite this, its components are linked to aspects mentioned in the description of intestinal health, either alone or in conjunction with other claims: the presence of OSs, particularly HMOs, probiotic microorganisms, and LA. Additionally, patents aiming for intestinal health and vision include DHA and ARA, as these compounds, as mentioned earlier, are crucial for neurodevelopment, including vision. These claims are inseparable, as good gastrointestinal health positively contributes to a balanced immune system that is neither hyper‐reactive nor apathetic, enhancing both overall immune defense and the recognition of non‐pathogenic substances and microorganisms.

#### 3.7.2 Growth

Various scales exist for assessing overall infant development, with the Bayley Scales of Infant and Toddler Development being the most pertinent and widely utilized. The fourth edition (Bayley IV), released in 2019, represents the latest interaction, though many studies still reference the third edition (Bayley III) from 2006. This assessment tool encompasses five scales: Cognitive, Language, Motor, Socio‐Emotional, and Adaptive Behavior. Physical growth and development are categorized under the Motor scale, while guidelines from the European Society for Paediatric Gastroenterology Hepatology and Nutrition are commonly applied for assessing infant physical parameters such as length (or height), weight, and head circumference (Romano et al. 2017; Gould et al. 2024; Stephenson et al. 2024).

Literature consistently suggests correlations between minerals such as potassium, magnesium, calcium, iodine, manganese, zinc, and infant growth and development. However, these studies are often constrained by significant limitations, necessitating further research to establish causal relationships between supplementation and adequate development (Sun et al. 2024; Azad et al. 2024). According to a systematic review by Azad et al. (2024), protein in breast milk is positively associated with infant length but not weight. Carbohydrates in breast milk have been found to positively influence infant weight, whereas the impact of breast milk fat on infant growth remains inconclusive due to methodological challenges.

Overall, scientific evidence concerning the relationship between breast milk composition and infant growth remains sparse. Consequently, infant formulas strive to replicate breast milk's macro and micronutrient profiles, as well as its bioactive components, under the assumption that breast milk is crucial for optimal child development and that similar nutritional benefits can be achieved until further research clarifies these relationships. Therefore, it is essential that infant formulas provide proper nutrition, which typically suffices for meeting growth criteria in most children, pending more definitive studies.

Growth is the third most prevalent claim, comprising 16% of the total. It is notable that this article did not specify the minerals in each patent due to their presence across all patents. Patents containing only minerals as additives were categorized as fortified milk and excluded during the eligibility assessment phase.

Patents focusing exclusively on growth typically include hydrolyzed proteins to aid in digestion, given the well‐established structural, regulatory, and carrier roles of proteins in the body. Huang et al. (2025) demonstrates the relation between the consumption of total or partially hydrolysed proteins and growth, showing the same potential for growth with less adverse events, such as allergies, when combined with vitamins, minerals, lipids, and carbohydrates in a formulation resembling breast milk, they are likely to support robust physical development in infants. Notably, as physical development involves both organ and brain growth, formulations targeting both growth and brain development often share similar active compounds, with those aimed at brain development potentially emphasizing neural connectivity, such as ARA, DHA, LA, and SA. The only patent also focused on brain development includes choline, which will be discussed in the next topic.

#### 3.7.3 Brain Development

Diet, during the first year of a newborn's life, plays a crucial role in ensuring proper growth and brain development of a child (Maldonado‐Pereira et al. 2023). According to Hosseini et al. (2024), early childhood nutrition can help the central nervous system grow, develop, and function. As various micro‐ and macronutrients, including vitamins, are cofactors in many ways and cycles, they also affect neurodevelopment. Consequently, dietary deficiencies in these nutrients can interfere with the normal process of neonatal brain development; and it is also essential to eliminate these deficiencies that can cause disorders (Li and Wang 2025).

Breast milk contains a variety of nutrients important for health and neurodevelopment that differ from formula and may contribute to the cognitive and other benefits seen in breastfed infants (Kumar and Kumar Thangamani 2026). Recently, interest has been drawn to MFGM, which occurs naturally as the fat globule membrane in breast milk, but is present only in trace amounts in most infant formulas where the source of fat is mainly vegetable oils (Yang et al. 2025). It has become possible to produce MFGM of sufficient quality and quantity to be included in infant formula, and studies have indicated that MFGM supplementation derived from bovine milk was safe and may benefit cognition (Gould et al. 2024).

Including components from bovine milk fat globules in infant formulas may support healthy brain development (Hedrick et al. 2021). Studies have shown that supplementing infant formulas with bovine MFGMs can substantially reduce the difference in health outcomes between formula‐fed and breastfed babies. It was noted that consumption of a formula supplemented with a lipid‐rich MFGM concentrate between 2 and 6 months of age improved cognitive performance and decreased infectious morbidity up to 6 months of age, close to that of breastfed infants (Timby et al. 2017).

In this way, the long‐chain polyunsaturated fatty acids, DHA and ARA, are considered essential for the maturation of the developing brain, retina, and other organs in newborns (Jasani et al. 2017). Infants who received formula with added bovine MFGM and LA in infant formula showed an accelerated neurodevelopmental profile, improved language subcategories, an association with age‐appropriate growth, and significantly less diarrhea and adverse events associated with breathing (Li et al. 2019).

#### 3.7.4 Vision

The development of the visual system begins immediately after birth and is driven by visual stimuli and interactions with the surrounding environment. This process occurs in parallel with the child's overall development, including neuropsychomotor progression, visuomotor coordination, cognitive abilities, as well as behavioral, environmental, and sociocultural adaptation. Normal visual development follows the broader trajectory of neurosensory maturation, with visual function progressively refined through continuous stimulation. The maturation of vision depends on adequate visual input, provided both by the environment in which the child is immersed and by interactions with caregivers (Zimmermann et al. 2019).

According to Spencer et al. (2024), human babies are completely dependent on nutrition from caregivers for basic survival and healthy development over a long period. Babies’ morphology has evolutionary adaptive value in its ability to trigger this nurturing care. A fundamental aspect of nurturing and sensitive care involves promptly and appropriately noticing children's signals and needs.

Elmlinger et al. (2026) indicates that throughout the first months of development after birth, the baby demonstrates social learning related to vision. A few hours after birth, newborns learn the skills of recognizing and identifying their mother's face. At seven weeks, eye contact begins to have social value in the interaction. There is an increase in the baby's gaze at the adult's face, the older the baby, between the first and third months of life. Therefore, upon completing two months of life, the baby acquires in‐depth vision with the convergence of both eyes and begins to pay more attention to interacting with his mother, demonstrating the differentiation of his social behavior from his behavior in relation to objects and begins to present the smile directed at the other. At three months, babies are sensitive to interactions between three people mediated by the examiner's direct contact with them. During the fourth month, the literature emphasizes a leap in babies' perception of the external object (Belini et al. 2010).

#### 3.7.5 Allergy

Cases of food allergy have increased significantly over the past decade, becoming a public health problem in several regions of the world, associated with a negative impact on the quality of life of individuals and of the whole family (Vilar et al. 2020). Milk allergy is normally associated with an immunological event against a specific protein, but milk intolerance can result from a genetically determined inability to metabolize the milk sugar, lactose (Hao et al. 2025).

Food intolerances are a group of non‐immune‐mediated adverse reactions to foods that lead to digestive and extra‐digestive symptoms. Lactose intolerance is one of the most common forms of food intolerance and is caused by a total or partial dysfunction of the lactase enzyme, responsible for the digestion of sugar present in dairy products, and thus, its clinical manifestations basically involve the gastrointestinal system (Barros et al. 2023).

One of the main allergic manifestations in childhood is allergy to cow's milk protein. The most problematic milk allergens are the most abundant proteins in milk and include caseins and the whey proteins alpha‐lactalbumin and beta‐lactoglobulin. Therefore, they can have two types of manifestations: immediate or delayed (Mato et al. 2024). According to Silva et al. (2017), among the immediate manifestations frequently observed are: angioedema, urticaria, and allergic hyperemia, vomiting, diarrhea, cough, and wheezing. Late manifestations may include: regurgitation and vomiting compatible with gastroesophageal reflux disease, diarrhea, bloody diarrhea (compatible with colitis), proctitis, poor weight gain or loss, excessive crying and abdominal distension, difficulty in evacuation and atopic eczema. Food allergies in children can increase the chance of recurrent wheezing by 2.17 times (Simões et al. 2019).

In infants, cow's milk protein is the most common precipitating agent of food anaphylaxis. This occurs with the progressive increase in vascular permeability, in which relatively minor symptoms appear earlier and portend a potentially fatal condition if not reversed in time (Sarinho et al. 2017). Therefore, babies who are allergic to cow's milk protein and who are not being breastfed should consume formulas without the proteins mentioned above. These foods can be based on hydrolyzed milk protein, free amino acids, or isolated soy protein (only for children over six months if they do not have gastrointestinal problems). For babies fed through breastfeeding, the mother should avoid consuming dairy products. The use of formulas with hydrolyzed proteins is the most recommended substitute for babies with cow's milk protein allergy, helping to control symptoms, maintain nutritional status and improve future tolerance to this protein. Formulas with amino acids should be reserved for cases of intolerance to hydrolyzed formulas, multiple allergies or serious clinical conditions that cause significant nutritional impairment (Moura et al. 2022).

#### 3.7.6 Nutrition Only

Babies and children are the most vulnerable groups to micronutrient deficiency, given the high intake of vitamins and minerals they need to support their rapid growth and proper development (Singh et al. 2025). Breastfeeding is one of the most effective ways to guarantee a child's health and survival. However, contrary to WHO recommendations, less than half of babies under 6 months of age are exclusively breastfed (Pecev‐Marinković et al. 2024).

For Ren et al. (2024), breast milk contains a wide range of components that are considered the optimal source of nutrients for feeding infants. It provides numerous benefits, such as cognitive development, defense against pathogens, digestion and absorption of nutrients, and a reduced risk of chronic diseases in adulthood. However, several circumstances can make breastfeeding difficult, resulting in a significant number of infants around the world receiving partial or exclusive formula feeding. Therefore, it is crucial to develop an ideal infant formula that closely matches the composition, functional properties, and health‐based outcomes of breast milk to meet the healthy growth needs of infants (Mosca and Giannì 2017). In addition to matching the nutrient levels of breast milk, certain infant formulas are also fortified or enriched with bioactive compounds and functional ingredients, such as prebiotic OSs, LA, and MFGM. Furthermore, there is a growing focus on studying the potential biological functional effects of these components when added to infant formulas.

The addition of bovine‐derived MFGM in infant formula may better approximate the complex lipid composition of human milk because human and bovine milk are highly homologous (Hedrick et al. 2021).

Enzymes play important roles in baby food manufacturing, from improving the taste, solubility, and digestibility of nutrients in baby food. Enzymes have also been used to produce some functional ingredients that are added to baby foods. For example, proteases are used for the in vitro digestion of milk protein for use in infant formula (Chauvet et al. 2025). Lipases are used to produce pure concentrates of functional lipids, such as omega‐3 DHA, to fortify baby foods. Pure DHA concentrates are often added to infant formulas because they have unique roles in babies' brain and eye development. Additionally, several enzymes have also been used to produce lactose‐free foods for lactose‐intolerant babies (Perez et al. 2024).

## 4 Conclusion

This study analyzed innovation trends in infant formulas through patents filed between 2015 and 2025, based on the specific patent search strategy adopted in this study, revealing a landscape strongly dominated by China, which accounted for over 97% of the analyzed patents. Gut health emerged as the main focus of innovation, followed by immunity and growth, with formulations frequently incorporating bioactive components such as HMOs, probiotics, OPO, LA, hydrolyzed proteins, and essential fatty acids. Additionally, there is a diversification of protein sources, with cow's milk remaining predominant, alongside the use of alternative sources that may offer distinct functional properties and reduced allergenicity.

Despite these advances, important challenges remain, including the need for stronger scientific substantiation of health claims and limitations related to the bioavailability of certain compounds, such as carotenoids. The study highlights the importance of future clinical research to validate the efficacy of novel protein sources and bioactive ingredients, as well as the development of technologies to improve bioavailability and digestibility. Furthermore, the emergence of multi‐stage formulas represents a technological approach identified within the analyzed patents to address the nutritional needs of different infant populations.

These findings should be interpreted within the scope of the search strategy adopted, as the selected IPC classifications and search terms may have influenced the patent landscape identified in this study and may not capture all technologies or innovations relevant to infant formula development.

## Author Contributions

**Giovanna Ferreira Paiva dos Santos**: writing – original draft, methodology, data curation. **Maria Esther Leal da Silva**: writing – original draft, methodology, data curation. **Rodrigo Stephani**: writing – review and editing, investigation. **Ítalo Tuler Perrone**: writing – review and editing, funding acquisition, investigation. **Juliana de Carvalho da Costa**: conceptualization, writing – review and editing, funding acquisition, supervision.

## Conflicts of Interest

Declare any conflicts of interest, or state that there are none to declare. If there are COIs to declare, uploaded them in a separate file where requested in the submission form.

## Figures and Tables

**FIGURE A1:**
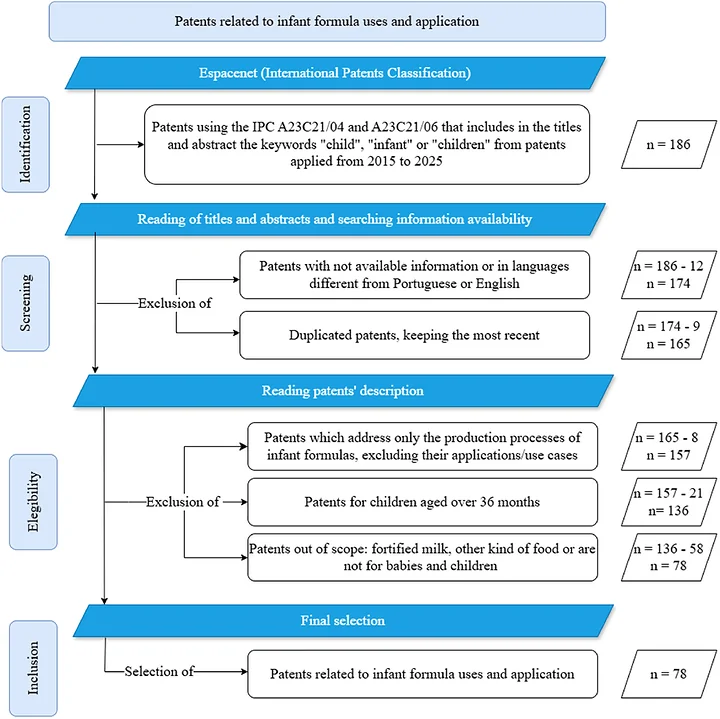
Research methodology flowchart: patents’ identification followed by screening and eligibility and inclusion criteria application.

## 1

FIGURE A1
